# The effect of virtual reality on cognitive, affective, and psychomotor outcomes in nursing staffs: systematic review and meta-analysis

**DOI:** 10.1186/s12912-023-01312-x

**Published:** 2023-05-19

**Authors:** Defi Efendi, Renny Wulan Apriliyasari, Juliana Gracia Eka Prihartami Massie, Cho Lee Wong, Regina Natalia, Bejo Utomo, Chiyar Edison Sunarya, Efa Apriyanti, Kee-Hsin Chen

**Affiliations:** 1 School of Nursing, College of Nursing, Taipei Medical University, No. 250 Wuxing Street, Xinyi District, 11031 Taipei City, Taiwan; 2 Faculty of Nursing, Universitas Indonesia & Nursing Department - Universitas Indonesia Hospital, Jl. Prof. Bahder Djohan, UI Campus, Depok, West Java Indonesia; 3 Institut Teknologi Kesehatan Cendekia Utama Kudus, Jl. Lingkar Timur No.Km.5, Jepang, Kec. Mejobo, Kabupaten Kudus, Central Java Indonesia; 4 Nursing Department, Universitas Indonesia Hospital, Jl. Prof. Bahder Djohan, UI Campus, Depok, West Java Indonesia; 5 The Nethersole School of Nursing - The Chinese University of Hong Kong, Shatin, New Territories, Hong Kong; 6 School of Nursing, Mitra Bunda Health Institute, Jl. Raya Seraya Nomor No.1, Tlk. Tering, Kec. Batam Kota, Batam, Kepulauan Riau Indonesia; 7 Post-Baccalaureate Program in Nursing, College of Nursing, & Cochrane Taiwan, Taipei Medical University, No. 250 Wuxing Street, Xinyi District, 11031 Taipei City, Taiwan; 8 Department of Nursing, Evidence-based Knowledge Translation Center, & Research Center in Nursing Clinical Practice, Wan Fang Hospital, Taipei Medical University, Taipei, Taiwan; 9 School of Medicine, Faculty of Health and Medical Sciences, Taylor’s University, Subang Jaya, Malaysia

**Keywords:** Affective, Cognitive, Learning satisfaction, Nursing staff, Psychomotor, Virtual reality

## Abstract

**Background:**

In the healthcare systems of the world, reinforcing the competence and professionalism of nurses has become a concern. Gaining clinical nursing competence in the healthcare system requires more effort, and additional training is required. Medical education and training have begun using digital technologies, such as virtual reality (VR). The purpose of this research was to examine the efficacy of VR in terms of cognitive, emotional, and psychomotor outcomes and learning satisfaction in nurses.

**Method:**

The study searched eight databases (Cochrane library, EBSCOHost, Embase, OVID MEDLINE, ProQuest, PubMed, Scopus, and Web of Science) for articles that met these criteria: (i) nursing staff, (ii) any virtual reality technology intervention for education, all levels of immersion, [1] randomized control trial and quasi-experiment study, and (iv) published articles and unpublished theses. The standardized mean difference was measured. The random effect model was applied to measure the main outcome of the study with a significance level of *p <* .*05*. The I^2^ statistic assessment was applied to identify the level of heterogeneity of the study.

**Results:**

A total of 6740 studies were identified, of which 12 studies with 1470 participants met the criteria for inclusion. The meta-analysis showed a significant improvement in the cognitive aspect (standardized mean difference [SMD] = 1.48; 95% CI = 0.33–2.63; p = .011, *I*^*2*^ *=* 94.88%), the affective aspect (SMD = 0.59; 95% CI = 0.34–0.86; p < .001, *I*^*2*^ *=* 34.33%), the psychomotor aspect (SMD = 0.901; 95% CI = 0.49–1.31; p < .001, *I*^*2*^ *=* 80.33%), and learning satisfaction (SMD = 0.47; 95% CI = 0.17–0.77; p = .002, *I*^*2*^ *=* 0%) aspects of the groups that received the VR intervention compared to the control groups. Subgroup analysis found that dependent variables (e.g., level of immersion) did not improve study outcomes. The quality of evidence was low which is affected by major methodological issues.

**Conclusions:**

VR may favorable as alternative method to increase nurse competencies. Randomized controlled trials (RCTs) on larger samples are needed to strengthen the evidence for the effect of VR in various clinical nurse settings. ROSPERO registration number: CRD42022301260.

**Supplementary Information:**

The online version contains supplementary material available at 10.1186/s12912-023-01312-x.

## Background

Reinforcing the competency and professionalism of nurses has become an issue in healthcare systems around the world [2–4]. As professionals with whom patients spend their time the most [5], nurses make essential contributions to the positive experiences of the patients they care for [6]. There has been evidence that competent nurses have the ability to increase the quality of care [7] in terms of safety [8, 9], prevention of physical injury [10], respect toward cultural matters [11, 12], and patient satisfaction [13]. However, guaranteeing nurses’ clinical competence in healthcare systems requires more effort [14, 15]. To address this, more training for nursing staffs is necessary [9].

Medical education and training have begun using digital technologies, such as the virtual world [16, 17]. Although the definition of the virtual world varies, its presence and use has become a major component of education technology [18], which uses instructional digital software called virtual reality (VR) [19, 20]. The term VR in this study refers to the virtual world that presents various forms of simulation technology in nurse education [16].

Nurses are different from other medical professionals in terms of the uniqueness of their knowledge and the art they perform in nursing care [21]. There have been studies of the healthcare workforce in general [21–23], but those results do not represent the nursing profession in particular. Studies of the use of VR with nurses are scarce [22], and some studies involved student nurses [16, 23, 24]. The outcomes in terms of knowledge, performance, self-efficacy, and communication skills have been applied only to nursing students [23, 25, 26]. Kyaw and colleagues suggested a study to evaluate VR with outcomes, including attitude, satisfaction, and behavior change, in future research because the findings in those areas are still limited [27]. Hence, a systematic review to measure the effectiveness of VR on professional nurses requires immediate attention. This meta-analysis is deemed the first to be conducted on nursing staffs in clinical service.

Besides the differences in the study background, previous meta-analyses have focused only on measuring knowledge levels as outcomes [25]. Therefore, by involving extracted literature reviews from a large database, this study will contribute additional findings to the previous ones. Bloom’s taxonomy of cognitive, affective, and psychomotor domains [28, 29] was applied in this study to identify similar study outcomes. Through the research gap above, this study aims to [30] measured the effect of VR on cognitive, affective, and psychomotor outcomes in nursing staff, and [2] identified the components that affect the outcomes of VR used to train nursing staff.

## Methods

### Design, search strategy, and study selection

This study has been reported according to the preferred reporting items for systematic reviews and meta-analyses (PRISMA) guidelines [31], and it has been registered with PROSPERO (No: CRD42022301260). Studies were collected from eight databases (CENTRAL from Cochrane library, CINAHL from EBSCOHost, Embase, MEDLINE from OVID, ProQuest, PubMed, Scopus, and Web of Science). For articles collected, there is no time limit. Articles from inception until May 2022 were collected using keywords combinations presented in Additional File 1. Then, two independent researchers performed the study screening using EndNote X9 software. Any disagreements were resolved through discussion.

### Eligibility criteria

The inclusion criteria in this study were: (i) nursing staff, (ii) any virtual reality technology intervention for education, all levels of immersion, [1] randomized control trial and quasi-experiment study, and (iv) published articles. The exclusion criteria consisted of: (i) pre-post test study without control grup,(ii) insufficient data for analyses, and [1] conference proceedings, abstract only, book chapters, reviews, letters, and editorials.

### Intervention

The term virtual reality refers to the spatial system that represents the physical world [32]. The computer system in VR consists of input and output devices that separate and connect the user with the virtual world [33, 34]. Isolation in VR can lead to a sense of immersion and presence—concepts that define VR [35]. Immersion in the virtual world is the extent to which users feel part of that world in a multi-dimensional concept that includes telepresence [33]. VR can be displayed on various devices, such as computer monitor and three dimensional (3D) or two dimensional (2D) television [36, 37], and head-mounted displays (HMDs) [33]. The keyboard, mouse, and trackball are examples of haptic interfaces in everyday life [33]. Avatars are often used to represent users in such simulations for creating real experiences in a virtual environment [36]. The level of immersion is a technical manipulation that can be applied to a broad range of paradigms [38]. The standardized classification of VR levels is described as VR: low, VR: medium, and VR: high [38, 39]. Comprehensive definition of the VR concepts in this study was summarized in Additional File 2.

### Outcomes of the study and operational definitions

Bloom’s taxonomy was used as the framework for classifying the learning outcomes from the articles included in this study. Bloom’s taxonomy was developed as a tool for educators to classify learning objectives and skills for students (Larkin & Burton, 2008). In this approach, learning is categorized according to three taxonomic domains: the cognitive domain (knowledge), the affective domain (attitudes), and the psychomotor domain (skills) [28]. According to Benjamin Bloom and his colleagues, the cognitive domain refers to the ability to think and solve problems; the affective domain involves attitudes and value systems, and the psychomotor domain represents the ability to do things [40]. To simplify the definition, we use the original version of Bloom’s taxonomy Details of the definitions in Bloom’s taxonomy are Additional File 2 [28, 40–43].

### Data extraction

Two independent investigator (RN, and CE) performed data extraction from the included studies. Information gained from each study included the first author, year of publication, country, participants, education level, age, experience, intervention and control group, results, size, study design, sample size, and key findings. Any discrepancy was resolved through a thorough discussion with the main author of this study.

### Risk of bias in individual studies

Risk of bias was assessed using version 2 of the Cochrane risk-of-bias tool for randomized trial studies. For randomized control trials included, bias from the randomization process, the effect of assignment to intervention, missing outcome data, outcome measurement, and the selection of the results report have been identified following the Cochrane guidelines [44]. Two reviewers independently completed the assessment of the risk of bias. Any conflicts were resolved by a third reviewer. Furthermore, the quality of the quasi-experimental studies used in this study was assessed using the JBI systematic review assessment [45]. The JBI critical assessment checklist for quasi-experimental studies comprises nine questions to assess threats to internal validity, namely on variables, participants included, interventions used, measurements of outcomes, and statistical analysis (Additional File 3).

### Synthesis of results

The standardized mean difference was calculated using comprehensive meta-analysis (CMA) V.3 software to measure the main and additional research outputs in this study. The overall effect size was tested with the standardized mean difference (SMD) and determined by calculating the Z-statistic with a significance level of p < .05. A sensitivity analysis of publication bias was performed [46] to assess the robustness of the studies’ results [47]. The I^2^ statistical assessment was used to determine the level of heterogeneity of the study [48] and to compare the impact of treatment from different interventions [49]. The Egger test [50] and visual inspection of the funnel plot asymmetry [51] were used to assess publication bias.

Meta-regression analysis of secondary data from factors influencing heterogeneity was performed on subgroups to identify and control for heterogeneity. A subgroup analysis was carried out on factors that were thought to affect the homogeneity of the study. Because the focus of this study was on the benefits of VR interventions, the variables included in the subgroup analysis were the level of immersion, head tracking, study design, and intervention context variables. The level of immersion was coded as high, moderate, or low. Head tracking was categorized into no head tracking and head tracking. Furthermore, randomized clinical trials (RCT) and quasi-experiments are elements of study design and intervention context categorized as emergency response and not emergency response. Meanwhile, the variables of screen resolution, field of view, refresh rate, and stereoscopy/3D were omitted from the subgroup analysis because of insufficient information. Non-visual stimuli and interactivity variables were not analyzed because they showed the same conditions in all studies. The variables of total sessions of interventions and total duration were analyzed in minutes, which is a continuous variable with meta-regression analysis to determine its effect on the main outcome of this study.

## Results

### Search results

**Fig. 1:**
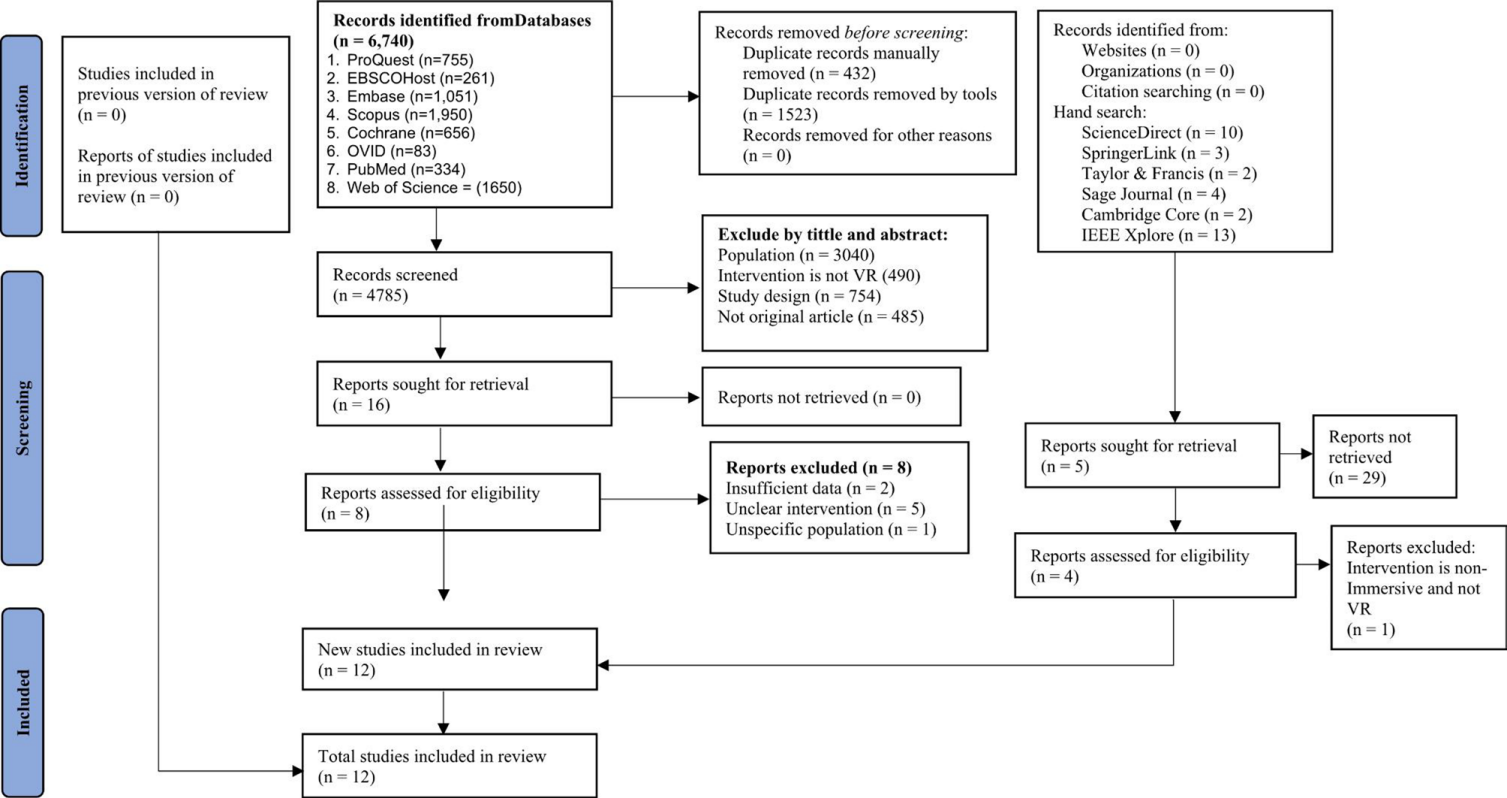
PRISMA Flowchart diagram of the study selection

A total of 6772 records were retrieved from 14 databases, and 432 duplicate records were removed using EndNote software. The final sample size was 12 studies comprising 7 RCTs and 5 quasi-experimental studies with full text for the systematic review and meta-analysis. Study screening and selection process shown as Fig. 1 [52].

### Study characteristics

Table 1 lists the characteristics of the 12 articles studied, which were published between 2002 [53] and 2021 [54–58]. Of these, four took place in China [53, 56–58], three in Taiwan [54, 55, 59], two in the United States of America [60, 61], and one each in Hongkong [62], Singapore [63], and Korea [64]. The participants in four studies were newly graduated registered nurses (NGRNs) [55, 56, 58, 59], in three they were registered nurses and enrolled nurses [53, 57, 62], and in five they were experienced RNs [54, 60, 61, 63, 64]. There was a total of 1470 nurses in the 12 studies. Brief explanation for VR were presented in the next Chapter (Table 2).

**Table 1:** Characteristics of the included studies

1st Author, Yr. (Country)	Participants	Study design	*n* M - Age (SD)		M – Experience (SD)		Procedural Setting	Intervention content	Comparator	Outcome	Measure
			I	C	I	C					
Pun, 2016 (Hong Kong)	Registered nurse and enrolled nurses	RCT, 2 arms	20 -	20 -	-	-	HD Unit	Procedural Skill and Specialty Care	Conventional training	Knowledge; skills competence	Self-developed instrument: HD knowledge test and HD skills competence checklist
Tsai, 2008 (Taiwan)	Novice nurses	RCT, 2 arms	11 22.6 (0.6)	12 22.7 (22.8)	0	0	Clinic	Procedural skills	Classroom teaching	Knowledge; clinical application; satisfaction in experimental group	Self-developed instrumentXXXeteriorons on knowledge, clinical applications, and satisfaction)
Wilfong, 2011 (USA)	Registered nurses	RCT, 2 arms	20 36.77 (-)	21 43.64 (-)	10.3 (-)	17.1 (-)	Large teaching hospital	Procedural skills	Traditional method of teaching	Number of tries to successfully insert an IV	Peripheral IV insertion survey
Zhang, 2021 (China)	Registered nurses	RCT, 2 arms	60 -	60 -	-	-	Emergency unit	Specialty care	Conventional training for 3 months	Capacity of emergency care; theoretical assessment; technical skills; preparedness for possible pandemic	Self-developed instrument (The emergency care capability rating scale; MCQ test for theoretical assessment; skill assessment tool); DPET
Chang, 2002 (China)	Registered nurses and enrolled nurses	RCT, 2 arms	14 -	14 -	11.71 (6.22)	10.14 (6.88)	Community	Procedural skills	Conventional teaching method using a plastic arm	Successful cannulation; anxiety level; intravenous cannulation performance; experience in VR learning	STAI; cannulation checklist; 4-question semi-structured interview to review the learning experience
Huang, 2021 (Taiwan)	Newly graduated registered nurses	Quasi-experiment, 2-group	38 26 (-)	36 25 (-)	0	0	Hospital	Procedural skill	Conventional flipped learning mode	Decision-making; learning achievement performance; problem-solving tendency; meta-cognition tendency; classroom engagement	Self-developed instrument (MCQ to evaluate knowledge; decision-making test rubric; the problem-solving tendency questionnaire; the meta-cognition tendency questionnaire; the classroom engagement questionnaire)
Liaw, 2015 (Singapore)	Registered nurse	RCT, 2 arms	35 25.58 (3.19)	35 -	-	-	Acute nursing care	Specialty care	No intervention	Performance; perception	RAPIDS; questionnaire survey on perception
Roh, 2013 (Korea)	Nurses	RCT, 2 arms	18 -	20 -	-	-	Hospital	Specialty care	Mannequin-based simulation with SimMan®	Know-ledge; self-efficacy; satisfaction	MCQs based on AHAACLFCQ; self-developed instrument to measure self-efficacy and satisfaction
Chang, 2021 (Taiwan)	Nursing staff	Quasi-experiment, 2-group	39 -	37 -	8.6 (-)	8.8 (-)	Chemotherapy unit	Specialty care	Traditional training approach	Learning achievement; learning attitudes; learning satisfaction; problem-solving skill	Self-developed instrument (learning achievement test, learning attitudes questionnaire, learning satisfaction questionnaire and eight cases for problem-solving skill test)
Green, 2017 (USA)	Registered nurse	Quasi-experiment, 2-group	17 -	15 -	-	-	Neonatal unit	Procedural skill	Live neonatal resuscitation simulations	Performance and retention of the neonatal resuscitation skills include: group function, preparation, communication, oxygen administration, ventilation, and chest compressions	The scoring tool for adherence to neonatal resuscitation guidelines
Luo, 2021 (China)	Newly graduated registered nurses	Quasi-experiment, 2-group	16 21.94 (1.29)	14 22.31 (0.70)	0	0	Hospital	Specialty care	Case study	Clinical judgment; self-confidence; satisfaction	Lasater clinical judgment rubric (LCJR); student self-confidence in learning scale; simulation design scale (SDS)
Zhong, 2021 (China)	Newly registered nurses	Quasi-experiment, 2-group	43 22.05 (0.82)	43 21.79 (0.72)	0	0	Emergency unit	Specialty care	Traditional learning methods	Emergency response ability; self-directed learning ability	AQCFCN-NED; RSSLCN

**Table 2:** Description of VR systems and interventions

1st Author, Yr. (Country)	Intervention	Immersion level	Intervention context	Device	Stereoscopy/3D	Head tracking	Non-visual stimuli	Intervention time (min)	Number of sessions	Theory using
Pun, 2016 (Hong Kong)	Web-based virtual training system	Low	Knowledge and skills competence about hemodialysis (HD)	Personal computer using internet browser connected with seven major catheter-access HD procedures	-	No	Audio	15 min	4 sessions	No
Tsai, 2008 (Taiwan)	Virtual reality computer simulation	Low	Learning Port-A Cath injection	3D computer graphicXXXeteriorateive circumscribed and external hardware controls linking to the desktop computer	Yes	No	Audio	40 min	6 sessions	No
Wilfong, 2011 (USA)	Virtual intravenous and patient simulator training	High	IV catheterization	Virtual IV Task Trainer and Nursing Anne Simulator (Laerdal Pty Ltd) with heptic interface	-	No	Haptic	1 h	1 session	No
Zhang, 2021 (China)	Combination virtual reality simulation training and technical skills training	Low	Emergency response of respiratory infectious disease	Virtual scene of different layout of zone and working environment	-	No	Audio	4 h	12 Sessions	No
Chang, 2002 (China)	Computer-based intravenous virtual training system (CathSim ITS)	Moderate	Training on intravenous cannulation	Computer, AccuTouch Tactile Feedback Device	-	No	Audio	User determined	User determined (in 1 week)	No
Huang, 2021 (Taiwan)	SVVR-EFL	High	Blood transfusion safety training	3D glasses, earphones, stereos	Yes	Yes	Audio	NR	NR	No
Liaw, 2015 (Singapore)	Web-based virtual simulation	Low	(30) understanding the underlying physiological signs of patient deterioration, (2) recognizing and managing deteriorating patients, and (3) communicating effectively about patient deterioration.	Web-based simulation of rescuing a patient in deteriorating situations (e-Rapids)	-	No	Audio	3 h	1 session	No
Roh, 2013 (Korea)	Computer based virtual simulation with MicroSim®	Low	Medical emergencies and advanced resuscitation training	MicroSim (Laerdal, Stavanger, Norway) in-hospital self-directed learning system	-	No	Audio	4 h	1 session	No
Chang, 2021 (Taiwan)	Experiential learning-based VR environment	High	The chemotherapy drug leakage accident protection	VR glass, Uptale VR Composer	Yes	Yes	Audio	50 min	1 session	Experiential learning theory
Green, 2017 (USA)	Independent computer-based virtual simulation scenario (eSim®)	Low	Neonatal resuscitation skills including airway, chest compressions, pulse oximetry, and communication	eSim® practice case	-	No	Audio	10 min	4 sessions	No
Luo, 2021 (China)	Web-based high-fidelity virtual simulator	Moderate	Simulation related to acute myocardial infarction, fracture of the lower leg, chronic obstructive pulmonary disease, and intestinal obstruction	SimMan 3G, vSim	-	No	Audio	1 h	User determined	NLN Jeffries simulation theory
Zhong, 2021 (China)	Flipped learning format combined with virtual simulation	Moderate	Emergency response ability training in nursing care of patients with anaphylactic shock, cardiac arrest, asphyxia, hypoglycemic coma	Obsim software	-	No	Audio	240 min	4 sessions	Self-regulated learning and socio-constructivist theories

### VR intervention

Table 2 provides detailed descriptions of the VR training. According to delivering method, three approaches to VR training used web-based simulation [56, 58, 63], seven used computer-based simulation [53, 57, 59–62, 64], and two used spherical video-based virtual reality (SVVR) simulation [54, 55]. The level of immersion of the VR training was low in six approaches [57, 59, 60, 62–64], moderate in three [53, 56, 58], and high in three [54, 55, 61]. The number of sessions ranged from 1 [54, 61, 63, 64] to 4 sessions [62], and the length of each session ranged from 10 min [60] to 4 h [57]. The total duration of training ranged from 1 h [61] to three weeks [59]. Three out of 12 studies had their interventions developed based on theoretical frameworks [54, 56, 58].

### Quality assessment

The bias assessment of seven RCT studies using the Cochrane risk of bias 2.0 instrument showed six studies [53, 57, 59–62, 64] were at high risk. These studies lacked detailed reporting of randomized and blind methods, but all studies reported complete data outcomes (Additional file 3). Meanwhile, four quasi-experimental studies using the JBI assessment tool showed that the results of four studies [56, 58, 63] were included in this systematic review and meta-analysis. All four studies reported having fully reported on the quasi-experimental method. Only one study (Green, 2017) did not fully report on follow-up data and similarities. Details of the quality assessment of quasi-experimental studies are provided in Additional file 3.

### Pooled results

#### The impact of intervention on the cognitive aspect

**Fig. 2:**
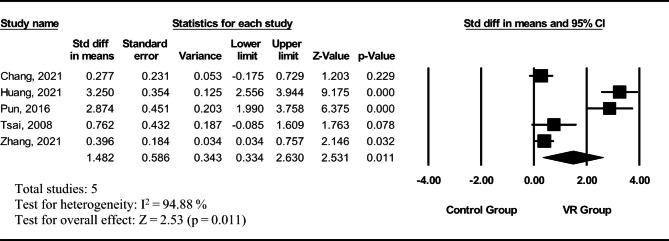
Forest plot of individual and combined effects from intervention reporting cognitive outcomes

The effects of VR interventions on the cognitive aspect among nurses were evaluated in five studies, and the pooled effects were statistically significant. As shown in Fig. 2, The effect on the cognitive aspect had a standardized mean difference (SMD) of 1.48 (95% CI = 0.33–2.63), and the studies were highly heterogeneous (I^2^ = 94.88%). Because of the small sample size, the moderator analysis (subgroup) was conducted only for the level of immersion. The moderator analysis showed no significant differences in effect sizes for the nurses’ cognitive aspect between the level of immersion (p = .788). The results of Egger’s test indicated that there was no publication bias (p = .162).

**Fig. 3:**
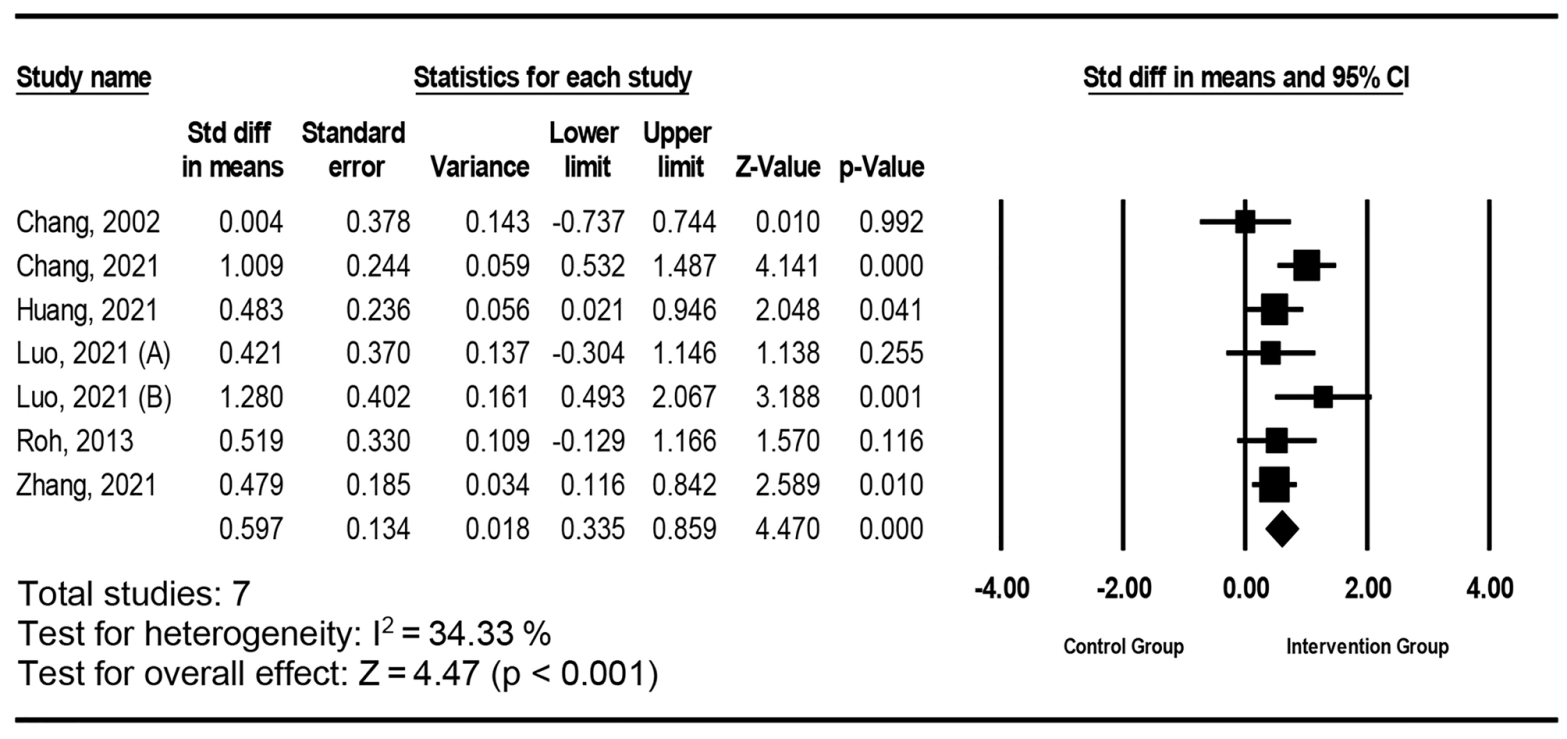
Forest plot of individual and combined effects from intervention reporting affective outcomes

#### The impact of intervention on the affective aspect

Figure 3 shows the effects of virtual reality interventions on affective among nurses in seven studies. This study found that the pooled effect size was statistically significant. The effect on affective had an SMD of 0.59 (95% CI, 0.34 to 0.86). The studies were moderately heterogeneous (I^2^ = 34.33%, p < .001). The moderator analysis showed no significant differences in effect sizes for nurse’s affective aspect between level of immersion (p = .713), study design (p = .060), and interventions context (p = .376). The results of Egger’s test indicated no publication bias (p = .462).

**Fig. 4:**
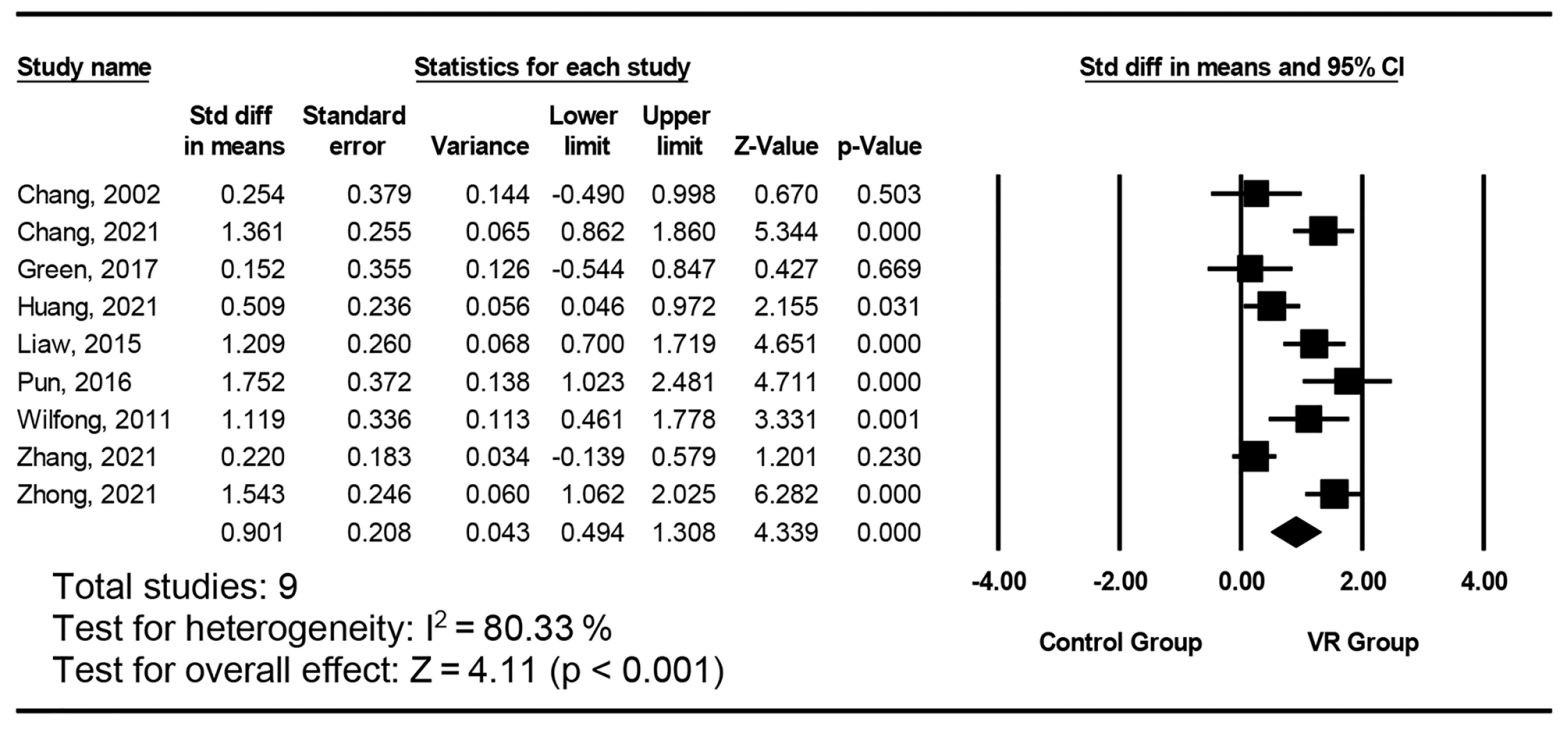
Forest plot of individual and combined effects from intervention reporting psychomotor outcomes

#### The impact of intervention on the psychomotor aspect

Figure 4 describes the effects of VR interventions among nursing staffs. The pooled results from nine studies indicated a statistically significant effect of VR intervention on the psychomotor aspect. The effect on psychomotor had an SMD of 0.901 (95% CI, 0.49 to 1.31). The studies were highly heterogeneous (I^2^ = 80.33%, p < .001). The moderator analysis showed no significant differences in effect sizes for nurses’ psychomotor ability between the level of immersion (p = .934). The results of Egger’s test indicated no publication bias (p = .462).

**Fig. 5:**
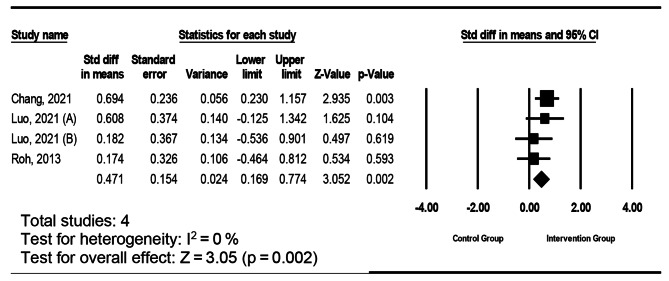
Forest plot of individual and combined effects from intervention reporting satisfaction outcomes

#### The impact of intervention on learning satisfaction

The effects of VR interventions on learning satisfaction among nurses were evaluated in four studies, and the pooled effect size was statistically significant. The effect on satisfaction had an SMD of 0.47 (95% CI, 0.17 to 0.77). Significant heterogeneity in the effect sizes of satisfaction was not found (see Fig. 5). the moderator analysis was not performed in this section.

**Table 3:** Moderator analysis: Subgroup analysis

Variable	n	SMD	95% CI	p	
**Cognitive**					
Level of immersion High Low	2 3	1.75 1.31	-1.16 to 4.67 -0.12 to 2.73	0.239 0.072	
Between sub-group p value = 0.788					
Head tracking Yes No	2 3	1.75 1.31	-1.16 to 4.67 -0.12 to 2.73	0.239 0.072	
Between sub-group p value = 0.788					
**Psychomotor**					
Level of immersion High Moderate Low	3 2 4	0.98 0.93 0.81	0.43 to 1.53 -0.33 to 2.19 0.09 to 1.53	< 0.001 0.028 0.148	
Between sub-group p value = 0.934					
Between sub-group p value = 0.572					
Head tracking Yes No	2 7	0.93 0.89	0.095 to 1.764 0.381 to 1.403	0.029 0.001	
Between sub-group p value = 0.940					
**Affective**					
Level of immersion High Moderate Low	3 3 2	0.74 0.58 0.49	0.23 to 1.26 -0.16 to 1.23 0.17 to 0.81	0.005 0.129 0.002	
Between sub-group p value = 0.712					
Study design RCT Quasi-experiment	4 3	0.43 0.91	0.19 to 0.68 0.48 to 1.35	0.001 < 0.001	
Between sub-group p value = 0.060					
Intervention context Emergency response Not emergency response	4 3	0.73 0.34	0.39 to 1.08 -0.08 to 0.74	< 0.001 0.105	
Between sub-group p value = 0.376					
Head tracking Yes No	2 5	0.74 0.51	0.23 to 1.26 0.19 to 1.84	0.005 0.002	
Between sub-group p value = 0.464					

RCT = randomized controlled trias

Table 3 describes the effect of the level of immersion, study design, use of head tracking, and the intervention context on cognitive, affective, and psychomotor outcomes. Subgroup analysis concluded that there was no effect of those independent variables on the study outcomes (p > .05). Meta-regression using a random effect model, in the Table 4, was used to examine the effect of total session and total minutes’ duration of intervention on the effect size of the cognitive, affective, and psychomotor aspects. Table 4 shows that those two covariates had no effect on the outcomes of the study (p > .05).

**Table 4:** Moderator analysis: Meta-regression analysis

Variable	n	β Coefficients	95% CI	p
**Cognitive**				
Total sessions of interventions Total duration in minutes	5 5	-0.104 -0.001	-0.454 to 0.246 -0.004 to 0.003	0.561 0.789
**Psychomotor**				
Total sessions of interventions Total duration in minutes	9 9	-0.054 -0.001	-0.157 to 0.049 -0.002 to 0.000	0.309 0.063
**Affective**				
Total sessions of interventions Total duration in minutes	7 7	-0.007 -0.002	-0.076 to 0.061 -0.001 to 0.001	0.839 0.578

### Sensitivity analysis

To assess the robustness of the results of the meta-analysis comparing the changes in cognitive, affective, and psychomotor aspects and learning satisfaction, sensitivity analyses were conducted by excluding one study at a time. No results were significantly altered, indicating the robustness of our results.

## Discussion

### Summary of key findings

This meta-analysis showed that all three domains of Bloom’s taxonomy, comprising cognitive, affective, and psychomotor aspects, were improved to a statistically significant level by the application of VR for training the nursing workforce. A significantly higher score for learning satisfaction in the VR groups also was revealed. In terms of moderator analysis, the level of immersion, study design, use of head tracking, and the intervention context, our moderator analysis found no significant difference in the effect sizes of the cognitive, affective, and psychomotor aspects in nurses. Finally, meta-regression also showed that interventions comprising total sessions and total minutes’ duration did not affect cognitive, affective, and psychomotor outcomes.

### Quality assessment

This VR study can be used as a reference with a low quality of evidence. Though the Egger’s test indicated no publication bias, high risk of bias was found in the reporting of RCT studies. Information on blinding or masking between the intervention and control groups was not available. The report of the randomization allocation technique was also not explained by the researchers. Not all RCT study protocols registered, leading to a lack of information for risk assessment of reporting bias. Prospective registration of clinical trials is important because of the issue of publication bias and selective reporting [65]. The publication status of the listed RCTs would provide clarity for readers to assess the research report [65]. The result of I^2^ also performed substantial heterogeneity among two outcomes. This may due to the variation of intervention, duration, and media used. Furthermore, this review also includes the four quasi-experimental studies which may interfer internal validity of the data pooling.

### Virtual reality and cognitive improvement

VR training considerably raised the cognitive level of the nursing staffs. Although they did not assess the cognitive aspect based on Bloom’s categories, previous studies have evaluated the effect of VR on knowledge outcomes as one part of the cognitive domain [43, 66]. This result is consistent with earlier reviews and meta-analyses that examined the impact of VR training and reported an increase in the applied knowledge of registered nurses and nursing students [67]. In addition, other studies focusing on nursing students revealed the same result [25, 68, 69]. The realism and immersion of the simulated VR world boosted pupil comprehension. Students believed that the ability to modify an avatar’s viewpoint enhanced their ability to learn [70]. On the other hand, VR showed more efficacy in nursing than conventional or other simulation-based education modalities. Virtual patients helped students to understand better the ideas taught and how to apply their new knowledge [71].

As evidenced by the previous study, Bloom’s taxonomy has provided a basis for learning in a VR environment [70]. Bloom’s taxonomy helps examine the process by which VR promotes knowledge acquisition. Bloom’s taxonomy has been extensively used in educational contexts to help students think and solve problems through the learning process. VR presents educational ideas of higher-level thinking in Bloom’s cognitive domains, such as creative and critical thinking, problem-solving, and multiple intelligences [70, 72]. It is also directly related to technological integration [70]. Bloom’s theory proposes that the acquisition of cognitive knowledge will proceed in three ways: comprehension, application, and analysis [43]. During VR simulations, the participants comprehend how to handle the problem in the most applicable method feasible, and they assess whether their knowledge is adequate to provide this clinical care [73]. VR programs may be essential for enhancing learning material as a supplement to conventional training [74].

### Virtual reality and affective improvement

Pooled data showed the effectiveness of VR in improving nurses’ affective aspect, compared to other traditional methods. This result is in line with a systematic review investigating the impact of VR intervention on nursing students’ and registered nurses’ emotional skills compared to other training method [75]. VR has the potential to foster empathy and help nurses visualize situations from the perspective of patients and in an affective domain [76]. Ouzouni and Nakakis [77] concluded that a nurse’s empathy is a two-pronged term that encompasses both emotional and mental reactions. Thus, using VR in education can improve nurses’ ability to detect another person’s emotions, comprehend their significance, and respond appropriately. A benefit of VR for influencing human emotions is that it simulates complex real-world situations [78].

According to Bloom’s taxonomy, in the affective domain, the behaviors of receiving and reacting must be used throughout the pre-simulation, pre-briefing or briefing, and participation phases [73, 79]. Previous research uncovered gaps and deficiencies in developing nursing students’ emotional domains for trust, decision-making, and patient care. The clinical simulation approach was planned and supported using Bloom’s taxonomy for competence building. The simulation linked with Bloom’s taxonomy might transcend the learning of cognitive and psychomotor domains, producing congruence between knowledge and the affective and psychomotor aspects in the nursing student [73, 80]. The affective domain is established during the first phases of the clinical simulation, when the person’s determination and drive to learn are appreciated and heightened during debriefing, which includes all the behaviors described by Bloom’s taxonomy throughout the reflective process. This supports the significance of debriefing for the development of clinical nursing competence [73].

### Virtual reality and psychomotor improvement

Though the included articles comprised a range of participants and types of psychomotor skills, this meta-analysis showed that VR intervention could improve the psychomotor domain in nurses. These results support the findings of several studies [67, 81, 82]. On the other hand, in a meta-analysis that encompassed nursing student participants, VR was not more effective than traditional methods in improving nursing skills [23]. This finding is consistent with other reviews that VR was not proven to influence skill development in nursing students and registered nurses [16]. From this point, it can be argued that the conclusions of some recent studies are inconsistent. This might be because there were various participant characteristics, such as years of experience and level of education. It cannot be overlooked that these variables affect the clinical skills of nurses.

In Bloom’s taxonomy, the psychomotor aspect is in the second phase of clinical simulation, which is initiated by the cognitive and affective domains in the first phase [83]. In other words, the performance of psychomotor skills is affected by the pre-knowledge and motivation of nurses, and these aspects are gained from the experience of environment exposure. Our analysis of the studies showed that VR significantly improved nurses’ cognitive and affective aspects. Thus, initiating psychomotor improvement in nursing staffs by the affective and cognitive aspects is guaranteed in this study. The role of VR is imperative to help nurses get closer to the real environment [84]. Thus, VR is presumed to provide positive benefits in improving clinical skills.

### Virtual reality and learning satisfaction

This review concluded that VR could significantly improve nursing staff’s learning satisfaction compared to other training modalities. Compared to the three domains of Bloom’s taxonomy, the number of included studies on learning satisfaction was relatively small. Nonetheless, the four included studies were remarkably similar. This finding is not supported by Chen, Leng [68] who found no significant increase in learning satisfaction among students of nursing and other health professions. However, it is essential to consider the homogeneity of immersion levels across studies, which is likely to influence the results.

Researchers have shown strong positive associations between student motivation and academic performance [85, 86]. Based on anatomical arrangement, Moro, Štromberga [87] discovered that one-third of participants found the VR approaches disorienting and annoying. Using VR may result in cybersickness, including nausea, dizziness, and headache. Thus, future research should concentrate on the detrimental impacts of VR, such as impaired vision and confusion [85, 88].

### Moderator analysis

The statistical test of moderator analysis of the subgroups of the categorical and continuous variables in the meta-analysis and meta-regression showed that there was no significant difference in the effectiveness of VR at various levels of attenuation (high, moderate, or low), the presence or absence of head tracking, study design (RCT or quasi-experimental), intervention context (emergency or not emergency), total sessions of interventions, and total minutes’ duration. The cognitive, affective, and psychomotor domains showed the same results from the moderator analysis. This finding is consistent with a previous meta-analysis, which reported that content covariates, level of immersion, length of sessions, and the number of sessions did not affect knowledge outcome scores [25]. However, it cannot be concluded that there is no effect of covariate variables on the effectiveness of VR because the studies included in this meta-analysis were mostly conducted on small samples, and the bias of most studies was assessed as high risk. According to Woon, a low to medium level of immersion is more effective in providing a learning environment than a high level of immersion [25]. Further exploration is needed to determine the effect of VR on the levels of immersion, interactivity [27], VR devices, and intervention context.

### Strengths and limitations

As far as the authors know, this study is the first to evaluate the effectiveness of VR in nursing staff populations. There was no publication bias from the 12 studies. This work provides three outcomes of VR intervention, which are inspired by Bloom’s taxonomy. The cognitive, affective, and psychomotor domains are deemed to be the pedagogic mechanism for the development of nursing competence in clinical settings [83]. Moreover, this work conducted a sensitivity analysis that showed the robustness of the results. However, risk of bias was high in most of the study included. The heterogeneity among two outcomes were considered substantial. In addition, the quasi-experiment method was still included in this review because of the lack of studies focused on nursing staffs. The other shortcomings were the exclusion of potential appropriate study related engineering area due to the conference proceedings were excluded in this study.Lastly, most of the analyzed studies were conducted on small sample sizes. Therefore, the analysis of study bias should be treated with careful caution.

### Impact on clinical practice training

This work strengthened the prospect of involving VR in training nurses and improving their nursing competency. There is high confidence in the effectiveness of VR in increasing the cognitive, affective, and psychomotor dimensions of nurses’ knowledge, which can lead to improved patient safety and increased patient satisfaction. Nevertheless, using VR has been presumed to be expensive and demanding. Fortunately, the literature has shown that VR has lower costs than traditional simulation [89]. Therefore, cost should not be a major concern of hospital management. However, technological issues may be a challenge for nursing departments. The use of VR should be understood comprehensively by the users so that the equipment is run properly. In addition, regular updates and maintenance of the programs are necessary to avoid glitches [90]. Thus, the existence of a special team that handles such technology is required.

## Conclusion and recommendations

This study provides evidence that VR is an effective alternative for improving nurses’ cognitive, affective, and psychomotor aspects and their learning satisfaction. Furthermore, this work found that there was no significance in effect size among dependent variables (e.g., level of immersion) did not improve study outcomes for all four outcomes. However, the possibility of heterogeneity and the risk of bias among studies cannot be ignored. Thus, the quality of evidence from this review was classified as low. Further RCTs with larger samples and robust methods based on the guidelines of the Consolidated Standards of Reporting Trials (CONSORT) are needed to ensure straightforward investigation of cause–effect relationships within the internal and external validity [91]. An evaluation of cost-effectiveness and technological feasibility is needed to guarantee the applicability of VR in settings with low resources. Further study should address the impact of VR technology on nurses’ clinical performance in real-world work settings.

## Electronic supplementary material

Below is the link to the electronic supplementary material.

Supplementary Material 1

Supplementary Material 2

Supplementary Material 3

Supplementary Material 4

Supplementary Material 5

## Data Availability

The supplementary materials for this study can be found in Additional file 1–5. Further inquiries should be directed to the corresponding author, and data from this study will be made available upon reasonable request.

## Abbreviations

VR: Virtual Reality
PRISMA: Preferred Reporting Items for Systematic Reviews and Meta-Analyses
CMA: Comprehensive Meta-Analysis
SMD: Standardized Mean Difference
RCT: Randomized Controlled Trial
HMDs: Head-Mounted Displays
POV: Field of View
3D: three dimensions
2D: two dimensions
CI: Confidence Interval
HD: Hemodialysis
NR: Not Report:IV:Intravenous
DPET: Disaster Preparedness Evaluation Tool
MCQ: Multiple-Choice Questions
CathSim ITS: CathSim Intravenous Training System
STAI: The State Trait Anxiety Inventory
SVVR-EFL: Spherical Video-Based Virtual Reality Based Experiential Flipped Learning
RAPIDS: Rescuing a Patient in Deteriorating Situation
AHAACLFCQ: American Heart Association Advanced Cardiac Life Support Course Questionnaire
SVVR: Spherical Video-Based Virtual Reality
HFS: High-Fidelity Simulation
VS: Virtual Simulation
LCJR: Lasater Clinical Judgment Rubric
SDS: Simulation Design Scale
AQCFCN-NED: Assessment Questionnaire of Clinical First-aid Capability of Nurses in Non-emergency Department
RSSLCN: Rating Scale of Self-directed Learning Competence for Nurses
CONSORT: Consolidated Standards of Reporting Trials
