# Supplementary material for: The effect of virtual reality on cognitive, affective, and psychomotor outcomes in nursing staffs: systematic review and meta-analysis
Source: BMC Nurs. 2023 May 19;22:170. doi: 10.1186/s12912-023-01312-x (PMC10197414; doi:10.1186/s12912-023-01312-x)
Supplement: Supplementary file 1 — Supplementary Material 1 [file 12912_2023_1312_MOESM1_ESM.docx]

**Additional file 1. Search Strategy**

| **No** | **Database/date** | **Keywords** |
| --- | --- | --- |
| 1 | Proquest/ Jan 7, 2022  Limit to:  Fulltext;  Source type: dissertations and theses, scholarly journals, working paper;  Document type: all;  Language: all | ((ab(nurs*) OR ab(nurse) OR ab(nursing staff) OR su(personnel, nursing) OR su(nursing personnel) OR su(registered nurses) OR su(nurse, registered) OR su(nurses, registered) OR su(registered nurse) OR su(nurse specialist)) AND (su(virtual reality) OR su(computer simulation) OR su(user-computer interface) OR ti(virtual realit*) OR ti(computer simulat*) OR ti(computer simulat*) OR ti(user-computer interface*) OR ti(augmented realit*) OR ti(artificial realit*) OR ti(simulated environment*))) OR ((ab(nurs*) OR ab(nurse) OR ab(nursing staff) OR su(personnel, nursing) OR su(nursing personnel) OR su(registered nurses) OR su(nurse, registered) OR su(nurses, registered) OR su(registered nurse) OR su(nurse specialist)) AND (ti(virtual environment*) OR ti(virtual patient*) OR ti(virtual hospital*) OR ti(virtual ward*) OR ti(virtual system*) OR ti(virtual setting*) OR ti(virtual simulat*) OR ti(virtual game*) OR ti(virtual gaming) OR ti(virtual world*))) OR ((ab(nurs*) OR ab(nurse) OR ab(nursing staff) OR su(personnel, nursing) OR su(nursing personnel) OR su(registered nurses) OR su(nurse, registered) OR su(nurses, registered) OR su(registered nurse) OR su(nurse specialist)) AND (ti(3D virtual world) OR ti(three-dimensional environment*) OR ti(three-dimensional setting*) OR ti(three-dimensional simulat*) OR ti(three-dimensional game*) OR ti(three-dimensional gaming) OR ti(three-dimensional world*) OR ti(3D environment*) OR ti(3D setting*) OR ti(3D simulat*))) OR ((ab(nurs*) OR ab(nurse) OR ab(nursing staff) OR su(personnel, nursing) OR su(nursing personnel) OR su(registered nurses) OR su(nurse, registered) OR su(nurses, registered) OR su(registered nurse) OR su(nurse specialist)) AND (ti(3D game*) OR ti(3D gaming) OR ti(3D world*))) |
| 2 | EBSCOhost/ Jan 8, 2022 | #S8: S5 OR S6 OR S7  #S7: S1 AND S4  #S6: S1 AND S3  #S5: S1 AND S2  #S4: TI three-dimensional gaming OR TI three-dimensional world* OR TI 3D environment* OR TI 3D setting* OR TI 3D simulat* OR TI 3D game* OR TI 3D gaming OR TI 3D world*  #S3: TI virtual ward* OR TI virtual system* OR TI virtual setting* OR TI virtual simulat* OR TI virtual game* OR TI virtual gaming OR TI virtual world* OR TI 3D virtual world OR TI three-dimensional environment* OR TI three-dimensional setting* OR TI three-dimensional simulat* OR TI three-dimensional game*  #S2: SU virtual reality OR SU computer simulation OR SU user-computer interface OR TI virtual realit* OR TI computer simulat* OR TI user-computer interface* OR TI augmented realit* OR TI artificial realit* OR TI simulated environment* OR TI virtual environment* OR TI virtual patient* OR TI virtual hospital*  #S1: AB nurs* OR AB nurse OR AB nursing staff OR SU personnel, nursing OR SU nursing personnel OR SU registered nurses OR SU nurse, registered OR SU nurses, registered OR SU registered nurse OR SU nurse specialist |
| 3 | Embase/ Jan 9, 2022 | #3: #1 AND #2  #2: 'virtual reality':ab,ti OR 'computer simulation':ab,ti OR 'user-computer interface':ab,ti OR 'virtual realit*':ab,ti OR 'computer simulat*':ab,ti OR 'user-computer interface*':ab,ti OR 'augmented realit*':ab,ti OR 'artificial realit*':ab,ti OR 'simulated environment*':ab,ti OR 'virtual environment*':ab,ti OR 'virtual patient*':ab,ti OR 'virtual hospital*':ab,ti OR 'virtual ward*':ab,ti OR 'virtual system*':ab,ti OR 'virtual setting*':ab,ti OR 'virtual simulat*':ab,ti OR 'virtual game*':ab,ti OR 'virtual gaming':ab,ti OR 'virtual world*':ab,ti OR '3d virtual world':ab,ti OR 'three-dimensional environment*':ab,ti OR 'three-dimensional setting*':ab,ti OR 'three-dimensional simulat*':ab,ti OR 'three-dimensional game*':ab,ti OR 'three-dimensional gaming':ab,ti OR 'three-dimensional world*':ab,ti OR '3d environment*':ab,ti OR '3d setting*':ab,ti OR '3d simulat*':ab,ti OR '3d game*':ab,ti OR '3d gaming':ab,ti OR '3d world*':ab,ti  #1: nurs*:ab,ti OR nurse:ab,ti OR 'nursing staff':ab,ti OR 'personnel, nursing':ab,ti OR 'nursing personnel':ab,ti OR 'registered nurses':ab,ti OR 'nurse, registered':ab,ti OR 'nurses, registered':ab,ti OR 'registered nurse':ab,ti OR 'nurse specialist':ab,ti |
| 4 | Cochrane / 15 Januari 2022  Limit: No | #1 = nurse OR nursing staff OR personnel, nursing OR nursing personnel OR registered nurses OR nurse, registered OR nurses, registered OR registered nurse OR nurse specialist  #2 = virtual reality OR computer simulation OR user-computer interface OR augmented reality OR artificial reality OR simulated environment OR virtual patient OR virtual patients OR virtual hospital OR virtual ward OR virtual system OR virtual systems OR virtual setting OR virtual settings OR  virtual simulation OR virtual game OR virtual games OR virtual gaming OR virtual world OR virtual worlds  #3 = 3D virtual world OR three-dimensional environment OR three-dimensional environmental OR three-dimensional setting OR three-dimensional settings OR three-dimensional simulation OR three-dimensional game OR three-dimensional games OR three-dimensional gaming OR three-dimensional world OR three-dimensional worlds OR 3D environment OR 3D setting OR 3D settings OR 3D simulation OR 3D game OR 3D games OR 3D gaming OR 3D world OR 3D worlds  #4 = #2 OR #3  #5 = #1 AND #4 |
| 5 | Web of science/ 15 Januari 2022  Limit to Article | #1 = AB = ('Nurs*' OR 'nurse' OR 'nursing staff' OR 'nursing personnel' OR 'registered nurse' OR 'nurse specialist')  #2 = AB=('virtual reality' OR 'computer simulation' OR 'user- computer interface*' OR 'augmented realit*' OR 'artificial realit*' OR 'simulated environment*'  OR 'virtual setting*' OR 'virtual simulat*' OR 'virtual game*' OR 'virtual gaming' OR 'virtual world*' OR '3D virtual world OR 'three-dimensional environment*' OR 'three-dimensional setting*' OR 'three-dimensional simulat*' OR 'three-dimensional game*' OR 'three-dimensional gaming' OR 'three- dimensional world*' OR '3D environment*' OR '3D setting*' OR '3D simulat*' OR '3D game*' OR '3D gaming' OR '3D world*') |
| 6 | Pubmed  / 15 Januari 2022  Limit= No  Limit = clinical trial, controlled trial, and randomized controlled trials = 27 | #1 = (((((((((("nurse"[Title/Abstract]) OR ("nursing staff"[Title/Abstract])) OR ("nursing personnel"[Title/Abstract])) OR ("personnel nursing"[Title/Abstract])) OR ("personnel nurse"[Title/Abstract])) OR ("personnel nurses"[Title/Abstract])) OR ("registered nurse"[Title/Abstract])) OR ("registered nurses"[Title/Abstract])) OR ("registered nursing"[Title/Abstract])) OR ("registered nurse personnel"[Title/Abstract])) OR ("registered nurse population"[Title/Abstract])  #2 = **(((((((((((((((((((((((((((((((((((((((((("virtual reality"[Title/Abstract]) OR ("virtual reality 3d"[Title/Abstract])) OR ("virtual reality airway simulator"[Title/Abstract])) OR ("virtual environment"[Title/Abstract])) OR ("virtual patient"[Title/Abstract])) OR ("virtual hospital"[Title/Abstract])) OR ("virtual ward"[Title/Abstract])) OR ("virtual system"[Title/Abstract])) OR ("virtual setting"[Title/Abstract])) OR ("virtual simulator"[Title/Abstract])) OR ("virtual simulators"[Title/Abstract])) OR ("virtual simulator training"[Title/Abstract])) OR ("virtual game"[Title/Abstract])) OR ("virtual gaming"[Title/Abstract])) OR ("virtual gaming simulation"[Title/Abstract])) OR ("virtual games"[Title/Abstract])) OR ("virtual gaming environment"[Title/Abstract])) OR ("virtual world"[Title/Abstract])) OR ("three dimensional"[Title/Abstract])) OR ("three dimensional based"[Title/Abstract])) OR ("three dimensional environment"[Title/Abstract])) OR ("three dimensional setting"[Title/Abstract])) OR ("three dimensional simulator"[Title/Abstract])) OR ("three dimensional game"[Title/Abstract])) OR ("three dimensional world"[Title/Abstract])) OR ("three dimensional worlds"[Title/Abstract])) OR ("3d"[Title/Abstract])) OR ("3d based"[Title/Abstract])) OR ("3d setting"[Title/Abstract])) OR ("3d environment"[Title/Abstract])) OR ("3d simulator"[Title/Abstract])) OR ("3d game"[Title/Abstract])) OR ("3d world"[Title/Abstract])) OR ("3d worlds"[Title/Abstract])) OR ("3d settings"[Title/Abstract])) OR ("3d simulation"[Title/Abstract])) OR ("3d games"[Title/Abstract])) OR ("computer simulation"[Title/Abstract])) OR ("user computer interface"[Title/Abstract])) OR ("computer simulator"[Title/Abstract])) OR ("computer simulation"[Title/Abstract])) OR ("user computer interfaces"[Title/Abstract])) OR ("augmented reality"[Title/Abstract])**  **#3 = #1 AND #2** |
| 7 | Ovid / 26 Januari 2022  Limit to: (full text and humans and randomized controlled trial) | Ovid MEDLINE(R) ALL <1946 to January 25, 2022>  1 (Nurs* or nurse or nursing staff or nursing personnel or registered nurse or nurse specialist).mp. [mp=title, abstract, original title, name of substance word, subject heading word, floating sub-heading word, keyword heading word, organism supplementary concept word, protocol supplementary concept word, rare disease supplementary concept word, unique identifier, synonyms]  2 (virtual reality or computer simulation or user-computer interface or virtual realit* or computer simulat* or user-computer interface* or augmented realit* or artificial realit* or simulated environment* or virtual environment* or virtual patient* or virtual hospital* or virtual ward* or virtual system* or virtual setting* or virtual simulat* or virtual game* or virtual gaming or virtual world* or 3D virtual world or three-dimensional environment* or three-dimensional setting* or three-dimensional simulat* or three-dimensional game* or three-dimensional gaming or three-dimensional world* or 3D environment* or 3D setting* or 3D simulat* or 3D game* or 3D gaming or 3D world*).mp. [mp=title, abstract, original title, name of substance word, subject heading word, floating sub-heading word, keyword heading word, organism supplementary concept word, protocol supplementary concept word, rare disease supplementary concept word, unique identifier, synonyms]  3 1 and 2 |
| 8 | SCOPUS  Jan 26, 2022 | ( TITLE-ABS-KEY ( nurs* ) OR TITLE-ABS-KEY ( nursing AND staff ) OR TITLE-ABS-KEY ( nursing AND personnel ) OR TITLE-ABS-KEY ( registered AND nurses ) OR TITLE-ABS-KEY ( registered AND nurse ) OR TITLE-ABS-KEY ( nurse AND specialist ) ) AND ( TITLE-ABS-KEY ( virtual AND reality ) OR TITLE-ABS-KEY ( computer AND simulat* ) OR TITLE-ABS-KEY ( user-computer AND interface* ) OR TITLE-ABS-KEY ( augmented AND realit* ) OR TITLE-ABS-KEY ( artificial AND realit* ) OR TITLE-ABS-KEY ( simulated AND environment* ) OR TITLE-ABS-KEY ( virtual AND environment* ) OR TITLE-ABS-KEY ( virtual AND patient* ) OR TITLE-ABS-KEY ( virtual AND hospital* ) OR TITLE-ABS-KEY ( virtual AND ward* ) OR TITLE-ABS-KEY ( virtual AND system* ) OR TITLE-ABS-KEY ( virtual AND setting* ) OR TITLE-ABS-KEY ( virtual AND simulat* ) OR TITLE-ABS-KEY ( virtual AND game* ) OR TITLE-ABS-KEY ( virtual AND gaming ) OR TITLE-ABS-KEY ( virtual AND world* ) OR TITLE-ABS-KEY ( 3d AND virtual AND world ) OR TITLE-ABS-KEY ( three-dimensional AND environment* ) OR TITLE-ABS-KEY ( three-dimensional AND setting* ) OR TITLE-ABS-KEY ( three-dimensional AND simulat* ) OR TITLE-ABS-KEY ( three-dimensional AND game* ) OR TITLE-ABS-KEY ( three-dimensional AND gaming ) OR TITLE-ABS-KEY ( three-dimensional AND world* ) OR TITLE-ABS-KEY ( 3d AND environment* ) OR TITLE-ABS-KEY ( 3d AND setting* ) OR TITLE-ABS-KEY ( 3d AND simulat* ) OR TITLE-ABS-KEY ( 3d AND game* ) OR TITLE-ABS-KEY ( 3d AND gaming ) OR TITLE-ABS-KEY ( 3d AND world* ) ) AND ( LIMIT-TO ( SUBJAREA , "MEDI" ) OR LIMIT-TO ( SUBJAREA , "NURS" ) OR LIMIT-TO ( SUBJAREA , "SOCI" ) OR LIMIT-TO ( SUBJAREA , "COMP" ) OR LIMIT-TO ( SUBJAREA , "HEAL" ) ) AND ( LIMIT-TO ( PUBYEAR , 2022 ) OR LIMIT-TO ( PUBYEAR , 2021 ) OR LIMIT-TO ( PUBYEAR , 2020 ) OR LIMIT-TO ( PUBYEAR , 2019 ) OR LIMIT-TO ( PUBYEAR , 2018 ) OR LIMIT-TO ( PUBYEAR , 2017 ) OR LIMIT-TO ( PUBYEAR , 2016 ) OR LIMIT-TO ( PUBYEAR , 2015 ) OR LIMIT-TO ( PUBYEAR , 2014 ) OR LIMIT-TO ( PUBYEAR , 2013 ) OR LIMIT-TO ( PUBYEAR , 2012 ) OR LIMIT-TO ( PUBYEAR , 2011 ) OR LIMIT-TO ( PUBYEAR , 2010 ) OR LIMIT-TO ( PUBYEAR , 2009 ) OR LIMIT-TO ( PUBYEAR , 2008 ) OR LIMIT-TO ( PUBYEAR , 2007 ) OR LIMIT-TO ( PUBYEAR , 2006 ) OR LIMIT-TO ( PUBYEAR , 2005 ) OR LIMIT-TO ( PUBYEAR , 2004 ) OR LIMIT-TO ( PUBYEAR , 2003 ) OR LIMIT-TO ( PUBYEAR , 2002 ) OR LIMIT-TO ( PUBYEAR , 2001 ) OR LIMIT-TO ( PUBYEAR , 2000 ) ) AND ( LIMIT-TO ( DOCTYPE , "ar" ) ) AND ( LIMIT-TO ( LANGUAGE , "English" ) OR LIMIT-TO ( LANGUAGE , "Chinese" ) ) AND ( EXCLUDE ( SUBJAREA , "SOCI" ) OR EXCLUDE ( SUBJAREA , "COMP" ) OR EXCLUDE ( SUBJAREA , "MATH" ) OR EXCLUDE ( SUBJAREA , "ENGI" ) OR EXCLUDE ( SUBJAREA , "BIOC" ) OR EXCLUDE ( SUBJAREA , "ENVI" ) OR EXCLUDE ( SUBJAREA , "PSYC" ) OR EXCLUDE ( SUBJAREA , "NEUR" ) OR EXCLUDE ( SUBJAREA , "BUSI" ) OR EXCLUDE ( SUBJAREA , "PHAR" ) OR EXCLUDE ( SUBJAREA , "ARTS" ) OR EXCLUDE ( SUBJAREA , "PHYS" ) OR EXCLUDE ( SUBJAREA , "MATE" ) OR EXCLUDE ( SUBJAREA , "CENG" ) OR EXCLUDE ( SUBJAREA , "DECI" ) OR EXCLUDE ( SUBJAREA , "CHEM" ) OR EXCLUDE ( SUBJAREA , "IMMU" ) OR EXCLUDE ( SUBJAREA , "DENT" ) OR EXCLUDE ( SUBJAREA , "ECON" ) OR EXCLUDE ( SUBJAREA , "ENER" ) OR EXCLUDE ( SUBJAREA , "VETE" ) OR EXCLUDE ( SUBJAREA , "EART" ) ) AND ( EXCLUDE ( LANGUAGE , "Portuguese" ) OR EXCLUDE ( LANGUAGE , "Spanish" ) OR EXCLUDE ( LANGUAGE , "French" ) OR EXCLUDE ( LANGUAGE , "German" ) OR EXCLUDE ( LANGUAGE , "Italian" ) OR EXCLUDE ( LANGUAGE , "Polish" ) ) AND ( EXCLUDE ( PUBYEAR , 2004 ) OR EXCLUDE ( PUBYEAR , 2003 ) OR EXCLUDE ( PUBYEAR , 2002 ) OR EXCLUDE ( PUBYEAR , 2001 ) OR EXCLUDE ( PUBYEAR , 2000 ) ) AND ( EXCLUDE ( PUBYEAR , 2005 ) ) |
