# Supplementary material for: The effect of virtual reality on cognitive, affective, and psychomotor outcomes in nursing staffs: systematic review and meta-analysis
Source: BMC Nurs. 2023 May 19;22:170. doi: 10.1186/s12912-023-01312-x (PMC10197414; doi:10.1186/s12912-023-01312-x)
Supplement: Supplementary file 2 — Supplementary Material 2 [file 12912_2023_1312_MOESM2_ESM.docx]

**Additional file 2. Blooms taxonomy and virtual reality concept**

**Definition of study’s outcome based on Bloom’s Taxonomy**

Bloom’s taxonomy is used as the framework for the classified outcome learning from articles included in this study. Bloom’s taxonomy was developed as a tool for educators to classify learning objectives and skills for students (1). Domain of learning is categorized according to the three taxonomic domains consisting of the cognitive domain (knowledge), affective domain (attitudes), and psychomotor domain (skills) (2, 3). According Benjamin Bloom and his colleagues, cognitive domain refer to the ability to think and solve problems, affective domain is attitudes and value systems, and the psychomotor domain known as the ability to do things (4). Table 1 explains in more detail the three learning domains.

**Table 1** Domain of learning

| **Domain of learning** | **Levels of teaching and learning** | **Definition** |
| --- | --- | --- |
| Cognitive domain  (5, 6) | Knowledge | “Involves the recall of specifics and universals, the recall of methods and processes, or the recall of a pattern, structure, or setting.” |
|  | Comprehension | “Refers to a type of understanding or apprehension such that the individual knows what is being communicated and can make use of the material or idea being communicated without necessarily relating it to other material or seeing its fullest implications.” |
|  | Application | “Use of abstractions in particular and concrete situations.” |
|  | Analysis | “Breakdown of a communication into its constituent elements or parts such that the relative hierarchy of ideas is made clear and/or the relations between ideas expressed are made explicit.” |
|  | Synthesis | “Putting together of elements and parts so as to form a whole.” |
|  | Evaluation | “Judgments about the value of material and methods for given purposes.” |
| Affective domain  (7) | Receptivity | “Refers to willingness to take interest in some phenomenon or something in the environment. Directs attention with the selective focus regarding the importance of the information.” |
|  | Response | “Refer to willingness participates in the activity, takes initiative, and is satisfied with the possibility to be active. Consciously reacts to the environment and expresses attention in a respective manner.” |
|  | Valuing | “Refers to the attachment of the value to some phenomenon, object, activity, etc. At this level a person’s attitude changes from a mere acceptance of the value to the commitment to comply with it.” |
|  | Organization | “A person arranges values, relates and synthesises them identifying priorities that help him/her solve internal moral conflicts.” |
|  | Internalization of values | “A person’s behaviour depends on the value system, and values control a person. When the behaviour consistently and for a long time is determined by the same values, a person develops the world view based on specific characteristics, and forms special individual learning ways.” |
| Psychomotor domain  (2, 4) | Perception | the ability to use sensory information to guide motor activity |
|  | Set | The readiness to act |
|  | Guided response | the early stages in learning a complex skill that includes imitation a displayed behavior and trial-and-error |
|  | Mechanism | The ability to convert learned responses have become habitual action and the movements can be performed with proficiency and confidence |
|  | Complex or overt response | The ability of skilfully of a complex movement pattern. |
|  | Adaptation | Skills are well developed and the fit special individual can modify movement patterns to requirements. |
|  | Originality (creation) | Creating new movement patterns for a specific situation or specific problem. |

**Definition of Virtual Reality Concept**

The term virtual reality refers to the spatial system that covers the physical world (8). The physical world is replaced with a virtual world that is created using a computer and provides access to interaction between individuals or groups (9). The computer system in VR consists of input and output devices that separate and connect the user with the virtual world (9). Isolation in VR will lead to a sense of immersion and presence as concepts that define VR. Immersion in the virtual world is the extent to which users feel part of the virtual world or also known as a sense of presence (9). The presence in question is the presence which is a multi-dimensional concept. This concept contains the meaning of telepresence. Telepresence occurs when users feel they are in an environment mediated (10) by virtual reality devices.

Virtual reality can be displayed on media categories in the form of (1) head-mounted displays (HMDs), which include mixed reality displays, (2) projection displays, which include flat-screen and curved-screen displays, and (3) monitors (9). A simple display of virtual reality in the form of a computer monitor or standard television based on considerations of model, brand, field of view specifications (POV), ratio, resolution, etc. (9). The screen display is in the form of 3D (11), 2D screen or speech for the type of object interaction in immersive virtual reality into a VR interface (12). Through the sense of touch, devices that allow users to study or interact with real environmental objects or virtual environments include Haptics. Keyboard, mouse, and trackball are examples of haptic interfaces in everyday life (9). While the haptic interface provides some tactile feedback to the user, the feedback they provide is indirect (9).

Virtual reality technology supports virtual simulations in creating real-life clinical scenarios where users can interact with the virtual world (13). Avatars are often used in virtual simulations as user representatives where the interaction method applied is two-way which serves as a tool to create real experiences in a virtual environment (11).

**LEVEL OF IMMERSION**

**Table 2** Examples of Virtual Environment Characteristics by Level and Aspect of Immersion

| **Level of immersion** | **Aspect of immersion** | | | | |
| --- | --- | --- | --- | --- | --- |
|  | **Inclusive** | **Extensive** | **Surrounding** | **Vivid** | **Matching** |
| Low | Numerous signals indicating the presence of device(s) in the physical world (e.g., use of a joystick or mouse to control the VE, direct instruction from an experimenter during the task) | Only accommodates 1 sensory modality (e.g., auditory, visual, motor/ proprioceptive); stimuli are not spatially oriented | Computer monitor presentation with limited field of view | Low fidelity and visual/color resolution; display may replicate features of the simulated environment, but not in a detailed or specific manner | No motion capture; visual experience does not match proprioceptive feedback |
| Moderate | Some signals indicating the presence of device(s) in the physical world (e.g., noise from a computer fan, weight and movement restriction from wearing a safety harness) | Accommodates 1–2 sensory modalities (e.g., auditory, visual, motor/ proprioceptive); stimuli may or may not be spatially oriented | Large-screen projection with extended field of view | Moderate fidelity and visual/color resolution; display replicates some features of the simulated environment, but some detail may be missing | Body segment motion capture (e.g., head, hand); visual experience somewhat altered to match proprioceptive feedback based on head or body segment movement |
| High | Limited signals indicating the presence of device(s) in the physical world (e.g., the weight of an HMD or an eye-tracking device) | Accommodates >2 sensory modalities (e.g., auditory, visual, motor/ proprioceptive); stimuli are spatially oriented | Head-mounted device or surround projection | High fidelity and visual/color resolution; display closely replicates multiple features of the simulated environment in great detail (e.g., correctly placed, dynamic shadows) | Full-body motion capture; visual experience altered to closely match proprioceptive feedback based on whole body movement |

HMD, head mounted device; VE, Virtual environment

Source: (14) (15)

**Table 3** Overall risk of bias analysis of RCT studies using ROB 2.0

| **No.** | **Study ID** | **Randomization process** | **Deviation from intented interventions** | | **Missing outcome data** | **Measurement of the outcome** | | **Selection of the reported results** | | **Overall** | |
| --- | --- | --- | --- | --- | --- | --- | --- | --- | --- | --- | --- |
| 1. | Chang 2002 | \|  \| \| --- \| | \|  \| \| --- \| | | \|  \| \| --- \| | \|  \| \| --- \| | | \|  \| \| --- \| | |  | |
| 2. | Liaw, 2015 |  |  | |  |  | |  | |  | |
| 3. | Pun, 2016 |  |  | |  |  | |  | |  | |
| 4. | Roh, 2013 |  |  | |  |  | |  | |  | |
| 5. | Tsai 2008 |  |  | |  |  | |  | |  | |
| 6. | Wilfong, 2011 |  |  | |  |  | |  | |  | |
| 7. | Zhang 2021 |  |  | |  |  | |  | |  | |
|  |  |  |  | |  |  | |  | |  | |
|  |  | = Low risk | |  | = Some concern | |  | | = High risk | |  |

**Table 4** Overall risk of bias analysis of quasi experiment studies using JBI

| **No** | **Question** | **Chang, 2021 (Taiwan)** | **Green, 2017 (USA)** | **Luo, 2021 (China)** | **Zhong, 2021 (China)** |
| --- | --- | --- | --- | --- | --- |
| 1. | Is it clear in the study what is the ‘cause’ and what is the ‘affect’ (i.e. there is no confusion about which variable comes first)? | Yes | Yes | Yes | Yes |
| 2. | Were the participants include in any comparisons similar? | Yes | Unclear | Yes | Yes |
| 3. | Were the participants included in any comparisons receiving similar treatment/care, other than the exposure or intervention of interest? | Yes | Yes | Yes | Yes |
| 4. | Was there a control group? | Yes | Yes | Yes | Yes |
| 5. | Were there multiple measurements of the outcome both pre and post the ntervention/exposure? | Yes | Yes | No | Yes |
| 6. | Was follow up complete and if not, were differences between groups in terms of their follow up adequately described and analyzed? | Yes | Unclear | Not Applicable | Not Applicable |
| 7. | Were outcomes of participants include in any comparisons measured in the same way? | Yes | Yes | Yes | Yes |
| 8. | Were outcomes measured in a reliable way? | Yes | Yes | Yes | Yes |
| 9. | Was appropriate statistical analysis used? | Yes | Yes | Yes | Yes |
|  | Overall apraisal | Include | Include | Include | Include |

**REFERENCES**

1. Larkin BG, Burton KJ. Evaluating a case study using Bloom's Taxonomy of Education. Aorn j. 2008;88(3):390-402.

2. Hoque ME. Three domains of learning: Cognitive, affective and psychomotor. The Journal of EFL Education and Research. 2016;2(2):45-52.

3. Nascimento J, Siqueira TV, Oliveira JLG, Alves MG, Regino D, Dalri MCB. Development of clinical competence in nursing in simulation: the perspective of Bloom's taxonomy. Rev Bras Enferm. 2021;74(1):e20200135.

4. Campos PRBd, Neto EdBC, Moreno UF. Proposal of a new taxonomy of the psychomotor domain for the engineering laboratory. 3rd International Conference of the Portuguese Society for Engineering Education (CISPEE)2018.

5. Armstrong P. Bloom’s Taxonomy: Vanderbilt University Center for Teaching; 2010 [cited 2022 5 August]. Available from: <https://cft.vanderbilt.edu/guides-sub-pages/blooms-taxonomy/>.

6. Bloom BS, Engelhart MD, Furst EJ, Hill WH, Krathwohl DR. Taxonomy of educational objetives: the classification of educational goals: handbook I: cognitive domain. New York, US: D. Mckay; 1956.

7. Savickienė I. Conception of Learning Outcomes in the Bloom's Taxonomy Affective Domain. Quality in Higher Education. 2010;7:37-59.

8. Dollinger N, Wienrich C, Latoschik ME. Challenges and opportunities of Immersive technologies for mindfulness meditation: A systematic review. Frontiers in Virtual Reality. 2021;2.

9. Human factors in simulation and training. New York: CRC Press 2008.

10. Lee H-G, Chung S, Lee W-H. Presence in virtual golf simulators: The effects of presence on perceived enjoyment, perceived value, and behavioral intention. New Media & Society. 2012;15(6):930-46.

11. Shin H, Rim D, Kim H, Park S, Shon S. Educational characteristics of virtual simulation in nursing: An integrative review. Clinical Simulation in Nursing. 2019;37:18-28.

12. Hepperle D, Weiß Y, Siess A, Wölfel M. 2D, 3D or speech? A case study on which user interface is preferable for what kind of object interaction in immersive virtual reality. Computers & Graphics. 2019;82:321-31.

13. Zhong MH, Jiang JX, Zhang H, Duan X. Combination of flipped learning format and virtual simulation to enhance emergency response ability for newly registered nurses: a quasi-experimental design. Interact Learn Environ. 2021:14.

14. Miller HL, Bugnariu NL. Level of immersion in virtual environments impacts the ability to assess and teach social skills in autism spectrum disorder. Cyberpsychology, Behavior, and Social Networking. 2016;19(4):246-2 56.

15. Kardong-Edgren S, Farra SL, Alinier G, Young HM. A call to unify definitions of virtual reality. Clinical Simulation in Nursing. 2019;31:28-34.
