# Supplementary material for: The effect of virtual reality on cognitive, affective, and psychomotor outcomes in nursing staffs: systematic review and meta-analysis
Source: BMC Nurs. 2023 May 19;22:170. doi: 10.1186/s12912-023-01312-x (PMC10197414; doi:10.1186/s12912-023-01312-x)
Supplement: Supplementary file 3 — Supplementary Material 3 [file 12912_2023_1312_MOESM3_ESM.docx]

**Additional file 3. Quality Assessment**

**Quality assessment for randomized control studies (R.o.B 2.0)**


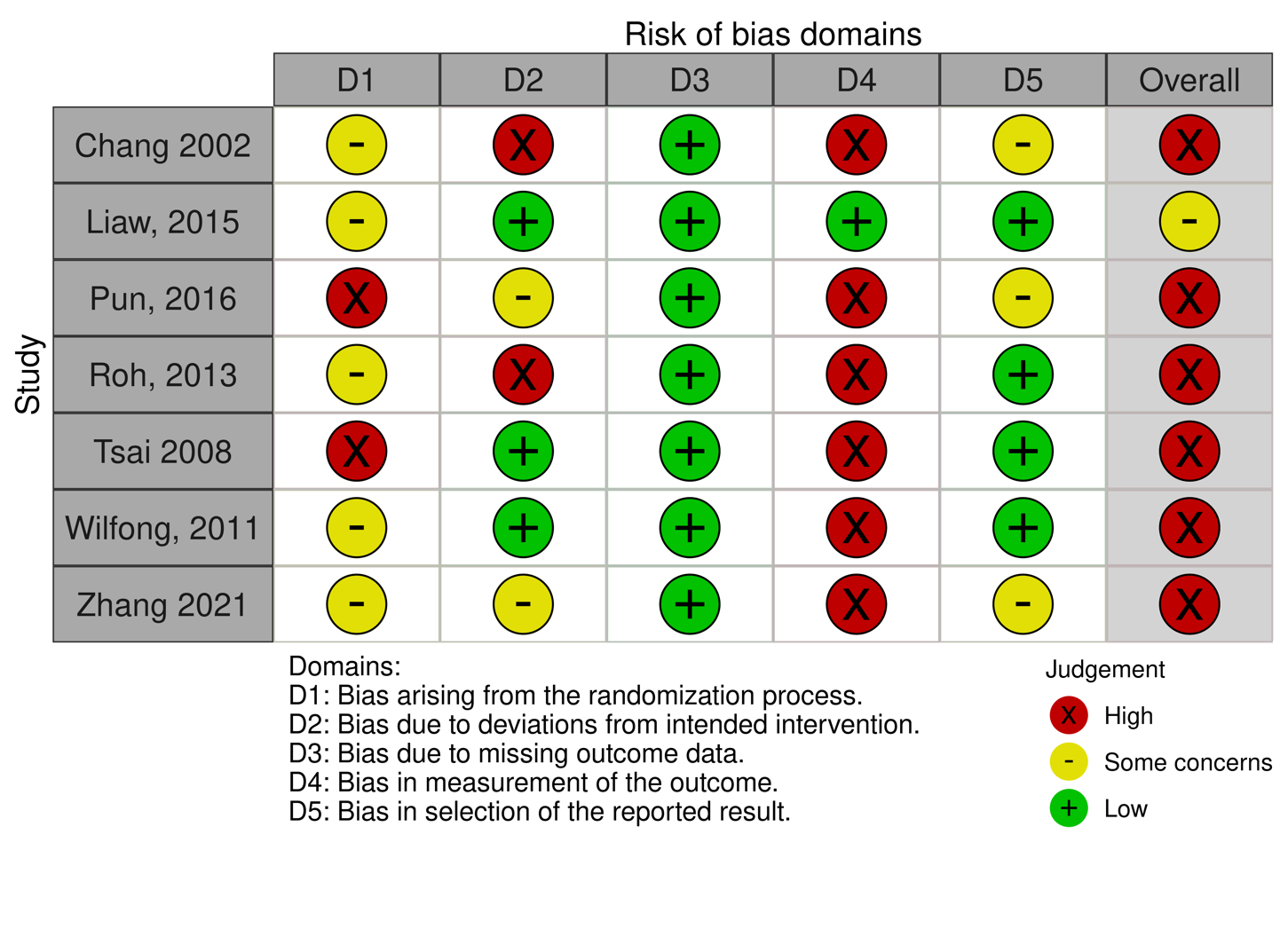


**Quality assessment for quasy-experimental studies (JBI Critical assessment Checklist for Quasi-Experimental Studies)**

| **No** | **Question** | **Chang, 2021 (Taiwan)** | **Green, 2017 (USA)** | **Luo, 2021 (China)** | **Zhong, 2021 (China)** |
| --- | --- | --- | --- | --- | --- |
| 1 | Is it clear in the study what is the 'cause' and what is the 'affect' (i.e. there is no confusion about which variable comes first)? | Yes | Yes | Yes | Yes |
| 2 | Were the participants include in any comparisons similar? | Yes | Unclear | Yes | Yes |
| 3 | Were the participants included in any comparisons receiving similar treatment/care, other than the exposure or intervention of interest? | Yes | Yes | Yes | Yes |
| 4 | Was there a control group? | Yes | Yes | Yes | Yes |
| 5 | Were there multiple measurements of the outcome both pre and post the interventuon/exposure? | Yes | Yes | No | Yes |
| 6 | Was follow up complete and if not, were differences between groups in terms of their follow up adequately described and analyzed? | Yes | Unclear | Not Applicable | Not Applicable |
| 7 | Were outcomes of participants include in any comparisons measured in the same way? | Yes | Yes | Yes | Yes |
| 8 | Were outcomes measured in a reliable way? | Yes | Yes | Yes | Yes |
| 9 | Was appropriate statistical analysis used? | Yes | Yes | Yes | Yes |
|  | Overall apraisal | Include | Include | Include | Include |
